# Mental Health, Well-Being, and Psychological Flexibility in the Stressful Times of the COVID-19 Pandemic

**DOI:** 10.3389/fpsyg.2021.647975

**Published:** 2021-05-17

**Authors:** Grażyna Wąsowicz, Szymon Mizak, Jakub Krawiec, Wojciech Białaszek

**Affiliations:** ^1^Department of Economic Psychology, Kozminski University, Warsaw, Poland; ^2^DecisionLab: Center for Behavioral Research in Decision Making, Institute of Psychology, SWPS University of Social Sciences and Humanities, Warsaw, Poland

**Keywords:** anxiety, depression, flourishing, mental health, psychological flexibility, stress, well-being

## Abstract

This study investigated the relationships between selected emotional aspects of mental ill-health (depression, anxiety, and stress, DASS) and mental well-health (well-being) experienced during the COVID-19 pandemic. The theoretical model of the study was based on Martin Seligman’s positive psychology and PERMA theory and Paul Wong’s Existential Positive Psychology 2.0 Theory, which postulates that negative experiences contribute to well-being and personal growth. The static approach was complemented by exploring the mediating role of psychological flexibility (defined as acceptance and action in the current situation) in the relationship between negative emotions and well-being. The data were collected during the initial phase of the COVID-19 pandemic from 277 participants (221 women), aged *M* = 33.83, SD = 12.77. The results confirmed that negative emotions correlated negatively with various domains of well-being (PERM), except for accomplishment (completing tasks and fulfill daily responsibilities). Moreover, negative emotions were related to the general well-being through psychological flexibility in that higher depression, anxiety, and stress were associated with lower psychological flexibility, which decreased general well-being. Finally, negative emotions were shown to be beneficial, having an adaptive effect that allows individuals to maintain their ability to cope with the situation, reach goals, and fulfill daily duties and responsibilities despite critical, stressful situation (like the COVID-19 pandemic) that limit their psychological flexibility. This observation confirmed the positive potential of negative aspects of life postulated within Existential Positive Psychology.

## Introduction

The study reported in the article was conducted in Poland in April 2020, one month after the first person infected with SARS-CoV-2 (March 4, 2020) was registered. At the beginning of April, the number of confirmed cases in Poland climbed to 2554, and deaths due to the COVID-19 infection reached 43. At the end of this month, the number of confirmed infections in Poland was 12,877, and 644 persons died. At the time of the manuscript revision (April 2021), 2.46 million Poles were infected, and 55 065 deaths were registered, with 132 million global infections and 2.87 million deaths.

The COVID-19 pandemic poses a serious threat to individuals’ well-being (Arslan et al., 2020a; Xiong et al., 2020; Yıldırım and Arslan, 2020), and it has caused many people to suffer from mental health problems worldwide (Arslan et al., 2020a; Qiu et al., 2020; Xiong et al., 2020). Recently published reports on the prevalence of depression, anxiety, and stress during the COVID-19 pandemic have shown high levels of mental health problems among health workers in Italy (for depression, anxiety, and stress, respectively: 24.73, 19.80, and 21.9%; Rossi et al., 2020; Yıldırım and Güler, 2021) and in Spain (46% in the case of depression, and 58.6% for anxiety; Luceño-Moreno et al., 2020). In the general Chinese population, 16.5% of participants reported high levels of depression, 28.8% reported anxiety, and 8.1% experienced high stress (Wang et al., 2020; Yıldırım and Arslan, 2020). The recent meta-analysis on depression, anxiety, and stress by Salari et al. (2020) confirmed high levels of mental ill-health in 1/3 of researched general populations. The analysis showed high levels of depression in 33.7% (based on 14 studies), anxiety in 31.9% (17 studies), and stress in 29.6% (five studies) of individuals. Therefore, factors that influence mental health and well-being in the times of the COVID-19 pandemic should be identified to support individuals and mental health services providers in their struggle against the negative psychological consequences of living in the stressful time (Tanhan et al., 2020). Arslan et al. (2020a) proposed that mental health consists of mental ill-health and well-health (see also Allen and McKenzie, 2015; Spiker and Hammer, 2019; Arslan and Allen, 2020). The former term refers to emotional states of depression, anxiety, stress, and adjustment problems that worsen optimal functioning, whereas the latter refers to fulfilling emotional, social, and psychological experiences (Arslan et al., 2020b).

In this study, we focused on the relationship between selected emotional aspects of mental ill-health (depression, anxiety, and stress) and mental well-health (well-being). The tripartite model of anxiety and depression (Clark and Watson, 1991; Lovibond and Lovibond, 1995; Brown et al., 1997) served as a theoretical background for the negative emotions investigated in the study. Within the model, depression is related to anhedonia and low or absent positive affect, anxiety is characterized by physiological hyperarousal and fearfulness, and stress is described as negative affect, persistent tension, irritability, and proneness to become upset (Lovibond and Lovibond, 1995; Brown et al., 1997). Concerning the well-being concept, we referred to the pillars of positive psychology founded by Martin Seligman, which try to determine the factors “that allow individuals, communities and societies to flourish,” experience well-being, and build individual strength (Seligman and Csikszentmihalyi, 2000, p. 5). Flourishing results from the interaction among five elements of well-being, namely, positive emotions, engagement in life, and work, relationships, meaning in life and work, and accomplishment (PERMA theory). Butler and Kern (2016), the authors of the PERMA profiler, defined these well-being dimensions (and the PERMA profiler scales) as follows. The positive emotions dimension refers to the general tendency to feel contentment and joy. Engagement in life and work means being absorbed, interested, and involved. The relationships dimension refers to human motivation to seek and maintain positive relationships, which expresses itself in feeling loved, supported, and valued by others. The meaning in life and work dimension refers to the sense of serving something “bigger” (Seligman, 2010), to a sense of a purposeful and valuable life worth living. The accomplishment dimension refers to the human motivation to achieve and master new skills and the feeling of being able to reach goals, complete tasks, and fulfill daily responsibilities. The PERMA model integrates three types of happiness, hedonic happiness (high positive affect and low negative affect), prudential happiness (engagement in life), and eudaimonic happiness (meaning in life and sense of fulfillment) (Wong, 2011, 2021). The model has been found to predict mental health (Kern et al., 2015; Butler and Kern, 2016).

The data reviewed above show that the COVID-19 pandemic may cause strong negative emotions expressed in depression, anxiety, and stress. Studies have reported negative relations between stress and well-being in various aspects of human life and clinical practice (Schönfeld et al., 2016; Wersebe et al., 2018). Anxiety and depression have also been found to lead to decreased well-being in various contexts (Smalbrugge et al., 2006; Lagnado et al., 2017; Malone and Wachholtz, 2018). Recent studies on the negative emotional states during the COVID-19 pandemic have shown negative correlations of stress (Bono et al., 2020), anxiety, and depression with psychological well-being (Vindegaard and Benros, 2020). However, to the best of the authors’ knowledge, no attempts have been made to describe the relationship between the negative emotional states and the well-being in terms of flourishing. To fill this gap, the first objective of this study was to explore the relationship between depression, anxiety, and stress and the five dimensions of flourishing. Based on conclusions from studies on negative emotions and psychological well-being, negative relationships could be expected.

On the other hand, negative emotions have an adaptive function (Nesse, 2019). The acceptance of negative emotions is important for optimal functioning (Stockton et al., 2019; Carreno et al., 2021), and the advantages of negative emotions have been documented in the literature (Calhoun and Tedeschi, 2006; Kashdan and Biswas-Diener, 2014; Ivtzan et al., 2015). Moreover, the Existential Positive Psychology 2.0 (Wong, 2011, 2020) stresses the importance of negative emotions and stressful experiences in individuals’ well-being. It postulates that “sustainable flourishing can only be achieved on the foundation of overcoming suffering” (p. 6) and that negative emotions experienced, e.g., in times of crisis, can lead to adaptive benefits, personal growth, and resilience. Since we did not have clear grounds for hypotheses, in the exploration, we limited ourselves to the research question about the existence and direction of relations between the three negative emotional states and the five dimensions of well-being, as defined in the PERMA theory. In particular, we were interested in finding out whether the negative emotional states experienced in the difficult time of the COVID-19 pandemic correlate with psychological benefits and mental well-health.

The traditional psychological approach offers several theories about what determines mental health and well-being. These include, e.g., references to (a) the intensity and quantity of positive compared with negative affective states (Diener, 2000; Fredrickson and Losada, 2005), (b) psychological needs of autonomy, competence, and relatedness fulfillment (Deci and Ryan, 2000), meaning in life (Arslan et al., 2020a), and (c) meaningful accomplishment (Csikszentmihalyi, 1990). The static approach taken in the abovementioned theories is criticized for not capturing “the dynamic, fluctuating, and contextually-specific behaviors that people deploy when navigating the challenges of daily life” (Kashdan and Rottenberg, 2010, p. 865). In the context of the COVID-19 pandemic, it is of utmost importance to identify psychological strengths that can help individuals overcome depression, stress, and anxiety and maintain well-being. Researchers have confirmed that meaning in life (Arslan et al., 2020a; Carreno et al., 2021; Eisenbeck et al., 2021), hope (Yıldırım and Arslan, 2020), positivity (Yıldırım and Güler, 2021), and self-efficacy (Yıldırım and Güler, 2020) positively influence mental health and well-being. They also identified two variables that cover dynamics in coping with adverse situations and relate to mental health and well-being. These are resilience, understood as one’s ability to recover from negative events and resist illness (Wong, 2020; Yıldırım and Arslan, 2020; Yıldırım et al., 2020), and psychological flexibility (Arslan et al., 2020a; Tanhan et al., 2020). We focus on the latter.

The term “psychological flexibility” covers many meanings, like adapting to situational demands, re-configuring mental resources, shifting perspectives, and balancing competing desires, needs, and life domains (Kashdan and Rottenberg, 2010, p. 865). Various facets of flexibility have been studied; however, it is believed that (a) psychological inflexibility and negative emotions correlate positively (Fresco et al., 2006; Tavakoli et al., 2019), (b) anxiety and depression are related to the loss of flexibility (Kashdan and Rottenberg, 2010) and are likely to appear when people need to adjust to changes in their environment (Mitchell et al., 2007; Mitchell et al., 2007; Mesidor and Sly, 2016) while coping flexibility leads to lower anxiety and depression (Cheng and Cheung, 2005), and (c) psychological flexibility has a major contribution to well-being (Kashdan and Rottenberg, 2010; Wersebe et al., 2018).

In the context of mental health threatened by the COVID-19 pandemic, Arslan et al. (2020b) found the relationship between coronavirus stress and psychological inflexibility. Moreover, psychological inflexibility mediated the relationship between coronavirus stress and mental ill-health, which means that negative emotions decreased psychological flexibility that in turn decreased mental well-health. Psychological flexibility measures how a person adapts to fluctuating situational demands (Kashdan and Rottenberg, 2010) and to the extent to which a person accepts the situational demands (Bond et al., 2011). According to Existential Positive Psychology, optimal functioning depends on the acceptance of negative emotions (Wong, 2011; Stockton et al., 2019; Carreno et al., 2021). Therefore, handling negative emotions with lower or higher acceptance and psychological flexibility affects individuals’ well-being.

Based on available results, one can generally hypothesize that higher levels of depression, anxiety, and stress decrease psychological flexibility (H1), which mediates the relationship between the negative emotional states and general well-being (H2). The psychological (in)flexibility consists of various psychological processes (Tanhan et al., 2020; see also Hayes et al., 2006; Tanhan, 2019), and well-being comprises various dimensions; therefore, we intended to explore the detailed relationships without posing detailed hypotheses, between the three emotional states and five dimensions of well-being and assess the mediating role of flexibility.

## Methods

### Participants

Three hundred and fifty-eight Poles participated in the study. To the methodological correctness, we excluded data from non-adults, those infected with the COVID-19, and those undergoing psychiatric treatment. The analyses were conducted on a sample of 277 participants (221 females and 56 males) aged 19–82 (*M* = 33.83, SD = 12.77). In the sample, 26% reported socioeconomic status higher than the average, 5.1% lower than average, and 69% claimed a medium status. The households consisted of one to nine members. The study presented here was a part of a larger project, and the goal was to obtain a broad and diverse research sample. No *a priori* power analysis was conducted. As an reviewer suggested, an a posteriori power analysis was conducted using a Monte Carlo power analysis for the indirect effects with a bootstrapped confidence interval. The analysis showed that for the collected sample, the power was 0.88 when assuming a small effect size (*r* = 0.25), which was appropriate for mediation analyses (Schoemann et al., 2017).

### Procedure

Participants were recruited by email and through social media (Facebook) from students and their social contacts using convenient sampling. The sample selection was not randomized. The study was conducted in accordance with the Declaration of Helsinki. Participation in the study was anonymous and voluntary, and the responses were confidential. Participants provided informed consent, which informed them that they could withdraw from the study at any time. They completed (in Polish) a series of questionnaires via the Google Forms platform, following all necessary demographic information, such as age, gender, place of residence, number of people in the household, and marital status. The participants also answered questions about their profession, the number and type of safety behaviors against infection, participation in psychiatric treatment, and socioeconomic status. On average, the procedure lasted about 20 min. Participants did not receive any incentives.

### Instruments

In this article, we present part of the study that used the following instruments.

**PERMA profiler** is a multidimensional scale that measures five domains of well-being, positive emotions, engagement, relationships, meaning, and accomplishment, with three items each (Butler and Kern, 2016). The total score for these domains constitutes a total measure of well-being. Participants expressed their answers on a Likert scale from 0 = not at all to 6 = completely (instead of the original 1 to 10, to be consistent with the other questionnaires in the entire set; see Dawes, 2008). The Cronbach’s alpha was 0.92 for the well-being measure overall, and 0.85 for positive emotions, 0.64 for engagement, 0.83 for relationships, 0.92 for meaning, and 0.80 for accomplishment subscales.

**Acceptance and Action Scale** (**AAQ-II**; Bond et al., 2011). The scale consists of seven statements measuring psychological flexibility. The participants assessed each statement on a 7-point scale (from 1 = “never true” to 7 = “always true”). The higher the overall score, the lower psychological flexibility (lower acceptance and action). For the study, we used the Polish version of the instrument (Kleszcz et al., 2018). The value of Cronbach’s alpha in the current study was 0.91.

**The Depression, Anxiety, and Stress Scale** (**DASS**-**21**; Lovibond and Lovibond, 1996; Brown et al., 1997) is a set of three self-reported scales and consists of 21 items assessing the symptoms of depression, anxiety, and stress experienced during the last week (in our study) on a 4-point scale ranging from 0 (did not apply to me at all) to 3 (applied to me very much, or most of the time). The scales provide three separate scores. Higher scores indicate more frequent symptoms of depression, anxiety, and stress. Cronbach’s alphas in our study were 0.91, 0.87, and 0.88 for each scale, respectively.

For this study, the first author adapted PERMA and DASS-21 scales to Polish, following the backtranslation procedure. The structure of the measures was confirmed in CFA (for PERMA: χ^2^ = 318.06, *p* < 0.001, for DASS-21 χ^2^ = 683.56, *p* < 0.001). Goodness of fit indices showed acceptable values (for PERMA: CFI > 0.91, for DASS-21: CFI > 0.86).

### Data Analysis

**FIGURE 1:**
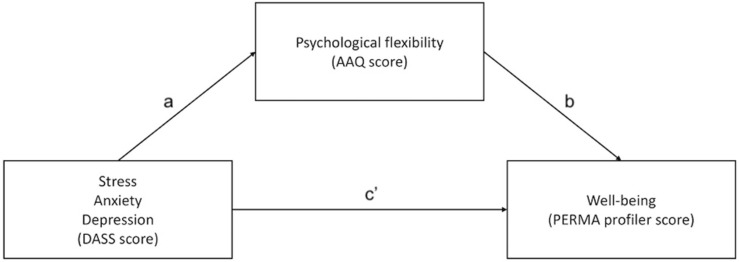
Model depicting the mediation of psychological flexibility on the relationship between negative emotions and well-being. Note: Paths a and b constitute the indirect effect, and path c′ the direct effect. The sum of all paths represents the total effect exerted on the dependent variable.

The data analysis was conducted using SPSS and PROCESS MACRO for SPSS (Hayes, 2017). The mediation analysis was chosen based on the theoretical status of psychological flexibility described in the introduction. We used bootstrapping procedures to test the significance of the mediation effects (5000 bootstrapped samples and 95% confidence intervals). These procedures were performed separately for each mediation analysis (not within a single model). The mediation model is depicted in Figure 1. The descriptive statistics (means and standard deviations) of all the measured variables are presented in Table 1.

**TABLE 1:** Descriptive statistics and distribution diagnostics for variables used in analyses.

	**PERMA PE**	**PERMA E**	**PERMA R**	**PERMA M**	**PERMA A**	**PERMA OA**	**AAQ**	**DASS S**	**DASS A**	**DASS D**
Mean	11.88	13.20	12.98	12.72	8.54	62.87	21.02	15.81	7.83	10.86
SD	3.19	2.97	3.73	3.94	3.55	13.44	9.32	10.67	9.28	10.67
Skewness	−0.75	−0.79	−0.81	−0.87	0.41	−0.79	0.58	0.48	1.63	1.15
Kurtosis	0.25	0.64	0.05	0.56	−0.57	0.52	−0.28	−0.62	2.41	0.59
Minimum	1.00	2.00	2.00	0.00	2.00	16.00	−	0.00	0.00	0.00
Maximum	18.00	18.00	18.00	18.00	18.00	88.00	−	42.00	42.00	42.00

## Results

In the first stage of the analyses, Pearson’s product-moment correlation coefficients were calculated to explore the relations between emotional scales (DASS-21) and well-being scales (PERMA, see Table 2). As expected, the relationships (from weak to strong) of depression, anxiety, and stress with the general well-being and most of the PERMA subscales were negative. However, a positive, strong relationship emerged between the DASS scales and the accomplishment scale.

**TABLE 2:** Pearson correlations between the results of the DASS-21 and PERMA scales.

	**Depression**	**Anxiety**	**Stress**
Positive emotions	−0.61***	−0.37***	−0.47***
Engagement	−0.40***	−0.17**	−0.21***
Relations	−0.38***	−0.15*	−0.19**
Meaning	−0.55***	−0.27***	−0.36***
Accomplishment	0.56***	0.52***	0.64***
Well-being	−0.60***	−0.30***	−0.37***

*p < 0.05, **p < 0.01, ***p < 0.001.

In the second stage, a two-step analysis was carried out to explore the relationship between well-being scales (PERMA), emotional scales (DASS-21), and psychological flexibility (AAQ-II scale). A partial correlation between emotional scales and well-being was calculated in the first step while controlling for psychological flexibility (Table 3). The results showed that when controlling for the psychological flexibility, the relation of anxiety and stress with general well-being and some of its subscales disappeared. Moreover, the relations between depression and general well-being and its subscales were weaker, but the signs of the relations remained the same (compared to the results presented in Table 2).

**TABLE 3:** Partial correlations between the results of the DASS and PERMA scales when controlling for psychological flexibility.

	**Depression**	**Anxiety**	**Stress**
Positive emotions	−0.43***	−0.14*	−0.23***
Engagement	−0.31***	−0.03	−0.07
Relations	−0.17**	0.09	0.09
Meaning	−0.32***	0.03	−0.04
Accomplishment	0.32***	0.31***	0.44***
Well-being	−0.40***	−0.02	−0.06

*p < 0.05, **p < 0.01, ***p < 0.001.

In the next step, we examined the mediating role of psychological flexibility in the relation between the DASS-21 and PERMA profiler scales. The mediation analysis showed a series of significant mediation effects (Table 4). In all cases, the path a between DASS-21 scales and AAQ score was significant (all *p*s < 0.001), and the coefficients were greater than zero (all estimates >0), indicating a direct relationship between negative emotions and psychological flexibility. Specifically, when negative emotions increased, people tended to manifest lower psychological flexibility. The result supported H1. Moreover, psychological flexibility was a significant mediator of the relationship between all the DASS-21 subscales and the overall PERMA profiler score, supporting H2. For stress and anxiety, full mediation effects occurred, while in the case of depression, the mediation effect explained only part of the relationship.

**TABLE 4:** The mediating effects of psychological flexibility on the relationship between the DASS-21 and PERMA profiler scales.

	**Stress**				**Anxiety**				**Depression**			
	**Estimate**	**95% CI lower**	**95% CI upper**	***p*-value**	**Estimate**	**95% CI lower**	**95% CI upper**	***p*-value**	**Estimate**	**95% CI lower**	**95% CI upper**	***p*-value**
**Positive emotions**												
Indirect effect	−0.07	−0.10	−0.05	***	−0.08	−0.11	−0.06	***	−0.05	−0.07	−0.03	***
a	0.51	0.42	−0.60	***	0.51	0.41	0.61	***	0.51	0.43	0.59	***
b	−0.14	−0.18	−0.10	***	−0.16	−0.20	−0.12	***	−0.09	−0.13	−0.06	***
Direct effect	−0.07	−0.11	−0.03	***	−0.05	−0.10	−0.01	*	−0.13	−0.17	−0.10	***
Total effect	−0.14	−0.17	−0.11	***	−0.13	−0.18	−0.08	***	−0.18	−0.21	−0.15	***
Proportion mediated	0.50	0.33	0.72	***	0.64	0.43	0.96	***	0.27	0.14	0.41	***
**Engagement**												
Indirect effect	−0.04	−0.06	−0.01	**	−0.04	−0.07	−0.02	**	−0.01	−0.03	0.02	ns
a	0.51	0.42	0.59	***	0.51	0.41	0.61	***	0.51	0.43	0.59	***
b	−0.07	−0.12	−0.03	**	−0.08	−0.12	−0.04	***	−0.02	−0.06	0.02	ns
Direct effect	−0.02	−0.07	0.02	ns	−0.01	−0.06	0.03	ns	−0.10	−0.15	−0.06	***
Total effect	−0.06	−0.10	−0.02	***	−0.05	−0.09	−0.01	**	−0.11	−0.15	−0.07	***
Proportion mediated	0.61	0.17	1.70	**	0.78	0.26	3.10	*	0.08	−0.12	0.35	ns
**Relationships**												
Indirect effect	−0.10	−0.13	−0.07	***	−0.10	−0.14	−0.07	***	−0.07	−0.10	−0.04	***
a	0.51	0.42	0.59	***	0.51	0.41	0.61	***	0.51	0.43	0.59	***
b	−0.20	−0.25	−0.14	***	−0.19	−0.24	−0.14	***	−0.13	−0.18	−0.08	***
Direct effect	0.03	−0.02	0.08	ns	0.04	−0.02	0.09	ns	−0.07	−0.12	−0.02	**
Total effect	−0.07	−0.11	−0.02	**	−0.06	−0.12	−0.01	*	−0.13	−0.17	−0.09	***
Proportion mediated	1.49	0.80	3.75	**	1.63	0.73	9.00	*	0.50	0.26	0.84	***
**Meaning**												
Indirect effect	−0.12	−0.16	−0.08	***	−0.13	−0.17	−0.09	***	−0.08	−0.11	−0.05	***
a	0.51	0.43	0.59	***	0.51	0.41	0.61	***	0.51	0.43	0.59	***
b	−0.23	−0.29	−0.18	***	−0.25	−0.29	−0.20	***	−0.16	−0.21	−0.11	***
Direct effect	−0.01	−0.06	0.03	ns	0.01	−0.04	0.06	ns	−0.12	−0.17	−0.08	***
Total effect	−0.13	−0.18	−0.09	***	−0.11	−0.17	−0.06	***	−0.20	−0.24	−0.16	***
Proportion mediated	0.89	0.63	1.31	***	1.12	0.73	1.85	***	0.40	0.26	0.59	***
**Accomplishment**												
Indirect effect	0.07	0.05	0.09	***	0.09	0.06	0.12	***	0.08	0.06	0.11	***
a	0.51	0.43	0.59	***	0.51	0.41	0.61	***	0.51	0.43	0.59	***
b	0.13	0.09	0.17	***	0.17	0.13	0.21	***	0.16	0.11	0.20	***
Direct effect	0.15	0.10	0.18	***	0.11	0.06	0.15	***	0.11	0.07	0.14	***
Total effect	0.21	0.18	0.24	***	0.20	0.15	0.25	***	0.19	0.15	0.22	**
Proportion mediated	0.31	0.20	0.45	***	0.44	0.31	0.61	***	0.43	0.29	0.60	***
**Over all**												
Indirect effect	−0.40	−0.53	0.28	***	0.42	−0.54	−0.30	***	−0.25	−0.36	−0.16	***
a	0.51	0.43	0.59	***	0.51	0.41	0.61	***	0.51	0.43	0.59	***
b	−0.78	−0.95	−0.61	***	−0.82	−0.98	−0.65	***	−0.49	−0.65	−0.33	***
Direct effect	−0.07	−0.22	−0.08	ns	−0.02	−0.20	0.14	ns	−0.50	−0.66	−0.37	***
Total effect	−0.47	−0.60	−0.33	***	−0.44	−0.63	−0.25	***	−0.75	−0.89	−0.62	***
Proportion mediated	0.85	0.58	1.20	***	0.96	0.65	1.53	***	0.33	0.20	0.49	***

All mediation analyses (except for depression and engagement) revealed significant indirect full or partial effects of the positive emotions, engagement, relationships, and meaning subscales of PERMA (all *p*s < 0.001 for indirect effects). The pattern of results was the same as described for the general PERMA score: depression, anxiety, and stress were negatively related to the well-being dimensions (estimates <0 for all direct effects) and positively related to AAQ-II score (all estimates >0 for path a of indirect effect), with the latter associated with lower well-being (all estimates <0 for path b of indirect effects). However, in the case of the accomplishment scale, the pattern of results was reversed. The AAQ-II score also mediated the relationship between the DASS-21 scales and the accomplishment scale (which were positively related with all estimates of direct effects >0). Furthermore, negative emotions were associated with lower psychological flexibility (path a estimates >0), and lower psychological flexibility was associated with a stronger feeling of being able to complete tasks and daily responsibilities (path b estimates >0).

## Discussion

In the study, we examined depression, anxiety, and stress experienced in the times of the COVID-19 pandemic and their relations with well-being. As expected, the results showed that negative emotions were negatively related to various domains of well-being (positive emotions, engagement, relationships, and meaning). We also observed positive relations between negative emotions and the accomplishment dimension of well-being. The accomplishment dimension refers to human motivation to achieve and master different skills, reach goals, complete tasks, and fulfill daily responsibilities; therefore, it reflects positive aspects of human functioning. The positive relationship between negative emotions and this dimension of well-being is consistent with the premise of Existential Positive Psychology, which postulates positive psychological benefits of negative, stressful aspects of life (Wong, 2011, 2020; Ivtzan et al., 2015).

In the study, we also found that higher depression, anxiety and stress were associated with lower psychological flexibility, defined as acceptance and action in a current situation. The results confirmed H1 and outcomes reported by other researchers, who showed that negative emotions are related to the loss of psychological flexibility. This observation is relevant to times before the pandemic (Kashdan and Rottenberg, 2010; Tavakoli et al., 2019) and during the pandemic (current study), suggesting that the relationship between negative emotions and psychological flexibility is universal. In the study, we also tested the mediating role of psychological flexibility in the relationship between negative emotions and well-being. We found that (a) depression, anxiety, and stress were related to the general well-being through psychological flexibility (H2 confirmed), and (b) lower acceptance and action (lower psychological flexibility) were associated with lower general well-being.

One new and interesting result referred to the relationship between negative emotions and the accomplishment dimension of well-being mediated by psychological flexibility. In everyday life situations, it is useful to seek psychological flexibility, benefits of which (just as the costs of psychological inflexibility) are well documented in the literature (Kashdan and Rottenberg, 2010). Moreover, psychological flexibility is seen as a protective factor against difficult situations (Masuda et al., 2010; Meyer et al., 2013; Kleszcz et al., 2018). The results of our study showed that negative emotions might decrease psychological flexibility, and although this lower psychological flexibility may reduce the general well-being, it also may give individuals a better sense of coping with a difficult situation by completing tasks and fulfilling daily responsibilities. In other words, negative emotions may be beneficial, having an adaptive effect that allows individuals to cope with the situation, reach goals, and fulfill daily duties and responsibilities despite the critical, stressful situations (like the COVID-19 pandemic) that limit their psychological flexibility. This observation confirms the positive potential of negative aspects of life postulated by Existential Positive Psychology (Wong, 2011, 2020; Ivtzan et al., 2015).

### Limitations and Implications

Due to the homogeneity of the sample (Polish participants), the generalizability of the results and external validity of the study are limited. However, the results suggest the importance of psychological flexibility for mental health in times of pandemic. Mental health professionals utilizing Acceptance and Commitment Therapy (ACT) could consider these results to help their clients build psychological strength by increasing psychological flexibility (Stockton et al., 2019; Arslan et al., 2020b; Tanhan et al., 2020).

Future research should verify the mediating role of psychological flexibility in the relationship between negative emotions and well-being in a longitudinal study and under different circumstances not affected by the COVID-19 pandemic. Especially, it is important to explore whether the mediating role of psychological flexibility in the relationship between the negative emotions and the accomplishment dimension of well-being reveals a universal pattern.

## Data Availability Statement

The raw data supporting the conclusions of this article will be made available by the authors, without undue reservation.

## Ethics Statement

The studies involving human participants were reviewed and approved by the Ethics Committee, Kozminski University. The patients/participants provided their written informed consent to participate in this study.

## Author Contributions

GW contributed to the manuscript by theoretical framing, coordinating the data collection, and manuscript writing. WB, SM, and JK conducted the analyses. GW, WB, SM, and JK wrote the first version of the manuscript. All authors revised the manuscript for important intellectual content and approved the final version to be published.

## Conflict of Interest

The authors declare that the research was conducted in the absence of any commercial or financial relationships that could be construed as a potential conflict of interest.
